# Inference of Kinetic Parameters of Delayed Stochastic Models of Gene Expression Using a Markov Chain Approximation

**DOI:** 10.1155/2011/572876

**Published:** 2010-12-15

**Authors:** Henrik Mannerstrom, Olli Yli-Harja, Andre S Ribeiro

**Affiliations:** 1 Computational Systems Biology Research Group, Department of Signal Processing, Tampere University of Technology, P.O. Box 553, 33101 Tampere, Finland; 2 Institute for Systems Biology, Seattle, WA 98103, USA

## Abstract

We propose a Markov chain approximation of the delayed stochastic simulation algorithm to infer properties of the mechanisms in prokaryote transcription from the dynamics of RNA levels. We model transcription using the delayed stochastic modelling strategy and realistic parameter values for rate of transcription initiation and RNA degradation. From the model, we generate time series of RNA levels at the single molecule level, from which we use the method to infer the duration of the promoter open complex formation. This is found to be possible even when adding external Gaussian noise to the RNA levels.

## 1. Introduction

Gene expression dynamics is influenced by even small fluctuations on the levels of various molecular species, such as RNA polymerases and transcription factors. In some cases, even the presence of a single molecule can cause phenotypic switching [1]. This makes the cellular metabolism inherently stochastic [2].

The stochasticity in the abundance of a substance is in general thought of being noise that obscures a signal that carries information relevant to the cell. However, recent evidence suggests that cells may be able to use the noise component in benefit of their survival [3]. Due to this, several modelling strategies have been proposed for accurately accounting for noise in the dynamics of gene regulatory networks (GRNs) [2, 4–7].

The chemical master equation is a probabilistic description of the dynamics of interacting molecules that fully captures the stochasticity of their kinetics. However, it is intractable to solve in the biologically relevant cases.

The stochastic simulation algorithm [8] (SSA) is a Monte Carlo simulation of the chemical master equation, allowing the study of complex models of gene expression. In the SSA, all chemical reactions are assumed instantaneous. However, several processes during the transcription and translation of a gene are highly complex, either involving many molecular species or involving reactions that are not bimolecular (e.g., the promoter open complex formation). To account for the effects of these events on the dynamics of RNA and proteins, the delayed SSA (DSSA) was proposed [5]. The ability of the DSSA to model chemical reactions with noninstantaneous events makes it a good tool to model GRN [6].

Assessing a model's accuracy and validity is important [9]. Even if experimental data has been used in model building, one must also be able to quantitatively rank the models based on the data. This ranking can be used to determine realistic parameter values, if these have not been measured directly, and to choose between models. As single molecule measurements of gene expression are becoming available [10], even the most detailed stochastic models can now be ranked.

Inference methods have been proposed to assess stochastic models of gene expression based on the SSA [11, 12]. Such methods are still lacking for the DSSA. Here, we present a method that, while requiring additional developments for analyzing complex gene networks, can be used to determine underlying features of single gene expression when simulated by the DSSA.

One feature in gene expression that has been proposed to influence noise in RNA and protein levels is the promoter open complex formation [13]. We use the proposed method to determine the duration of the promoter open complex formation from the dynamics of RNA levels of a delayed stochastic model of transcription.

## 2. Methods

### 2.1. Stochastic and Delayed Stochastic Simulation Algorithms

The Stochastic Simulation Algorithm (SSA) is a Monte Carlo simulation of the chemical master equation and, thus, is an exact procedure for numerically simulating the time evolution of a well-stirred reacting system [8]. Each chemical species quantity is treated as an independent variable, and each reaction is executed explicitly. Time is advanced by stepping from one reaction event to the next. At each step, the number of molecules of each affected species is updated according to the reaction formula.

For each reaction , the stochastic rate constant, , depends on the reactive radii of the molecules involved in the reaction and their relative velocities. The velocities depend on the temperature and molecular masses. After setting the initial species populations, , the SSA calculates the propensities , for all possible reactions, where  is the number of distinct molecular reactants combinations available at a given moment. Then, it generates two random numbers, , the time until the next reaction occurs, and , the reaction to occur. The probability for  is . Finally, the system time  is increased by , and the  quantities are adjusted to account for the occurrence of reaction , assuming it to be an instantaneous reaction. This process is repeated until no more reactions can occur or for a defined time interval.

Several steps in gene expression, such as transcripts assembly, are time consuming [14]. Such complex processes involve many reactions and events that cannot be modelled as uni- or bimolecular reaction events. To account for these events, the "delayed SSA" was proposed [5]. It uses a "waitlist" to store delayed output events. Multidelayed reactions are represented as . In this reaction,  is instantaneously produced and  and  are placed on a waitlist until they are released, after  and  seconds, respectively.

The delayed SSA proceeds as follows.

(1) Set , , set initial number of molecules and reactions, and create empty waitlist . Go to step (2).

(2) Generate an SSA step for reacting events to get the next reacting event  and the corresponding occurrence time . Go to step (3).

(3) Compare  with the least time in , . If  or  is empty, set: . Update the number of molecules by performing , adding to  both any delayed products and the time delay for which they have to stay in . This time can be chosen from a defined distribution. Go to step (4).

(4) If L is not empty and if , set . Update the number of molecules and , by releasing the first element in ; otherwise go to step (5).

(5) If , go to step (2); otherwise stop.

### 2.2. Delayed Stochastic Model of Transcription

A delayed stochastic model of transcription that includes the promoter open complex formation was proposed in Ribeiro et al. [6]. This model was shown to match the dynamics of transcription at the single RNA molecule level [15].

Our model is identical, except that it does not include an explicit representation of the RNA polymerase. This simplification is valid when the number of RNA polymerases does not vary significantly over time in the cell, which is likely to be the case in normal conditions in *E. coli* (Reaction (1)):(1)(2)

In Reaction (1),  (set to 1 in the begin of the simulation) is the promoter region of the gene while  is the stochastic rate constant of transcription initiation and its value is set to . This value assumes that the number of RNA polymerases available for transcription is always 40 [6] and that the binding affinity between RNA polymerase and transcription start site equals the one measured for the lac promoter [16]. The promoter delay, , is set to 40 s, in agreement with measurements for the lac Promoter [17]. Also, RNA stands for a fully transcribed RNA molecule, and  is the time that it takes for the transcription process to be completed, once initiated. This delay accounts for the promoter open complex formation (40 s), transcription elongation (mean value 60 s), and termination. Its value is randomly generated from a Gaussian distribution with a mean of 102 s and a standard deviation of 14 s. These values assume a lac promoter and a gene 2445 nucleotides long [16, 18].

Note that while Reaction (1) has a rate of , each activation cycle includes the open complex formation delay of  seconds, making the effective mean cycle duration equal to .

Reaction (2) models RNA degradation.  is the rate of degradation and is set to  (10 min mean lifetime), which is within realistic parameter values for *E. coli*[19].

**Figure 1:**
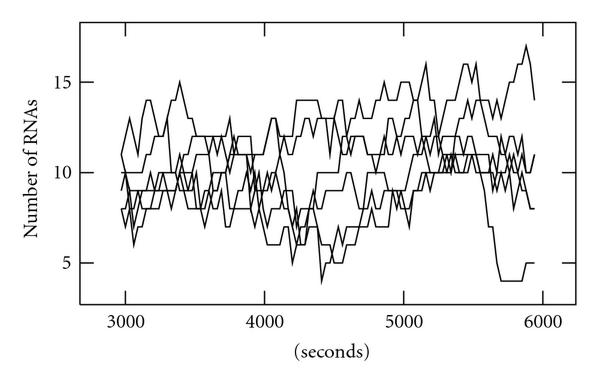
RNA levels from six independent simulations.

In Figure 1 are shown, as examples, levels of RNA molecules produced by independent simulations. The simulator ran for 6000 s from which the data from the last 3000 s was used as "steady state" data.

### 2.3. Approximative Inference

The system is approximated as a Markov chain with stationary distribution  and transition matrix . As we are only considering steady state conditions,  and  can be built by thoroughly sampling ( samples) from the simulated model. To compensate for the sampling error both  and  are "smeared out" with a kernel of . For example, if the raw sampling yields , then after the smearing , , .

The log likelihood  of the parameter , given a time series  can then be computed by(3)

where  is the RNA level at time .

The likelihood term is evaluated at suitable points over the full range of possible  values, ranging from zero to the maximum determined by dividing the mean RNA life time by the mean RNA level (in our case study, this ratio around 60). Due to the approximation of  and , the likelihood term will be nonsmooth and cannot be used as such. Instead, a quadratic polynomial is fitted to the point samples. The quadratic fit was chosen because it gives a likelihood proportional to a truncated normal distribution. Similar to the application of Bayes' theorem with a flat, non informative prior, the likelihood is converted to a probability distribution by normalizing it to unit probability.

### 2.4. Error Model

To simulate measurement error, normally distributed noise with zero mean and 0.5 standard deviation was added to the simulated time series used for inference. Any negative values were zeroed.

## 3. Results

In all simulations we set the sample interval to 30 s, as this is currently the shortest interval possible in real measurements of RNA numbers at the single molecule level [10]. The inference was made using these point samples.

**Figure 2:**
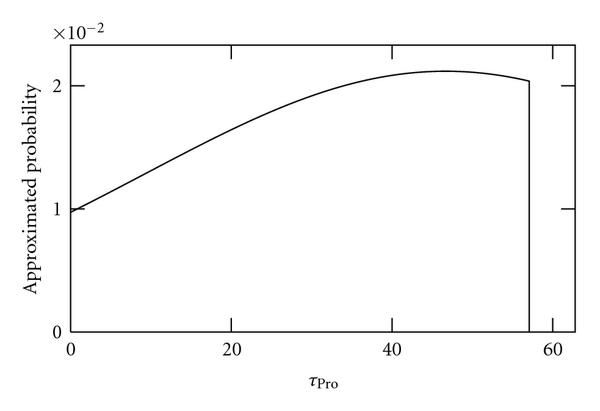
**Approximated probabilities for values of  inferred using simulated noiseless data from 10 cells**. The true value is 40, the maximum likelihood value is 46.7 and the expected value is 31.8.

**Figure 3:**
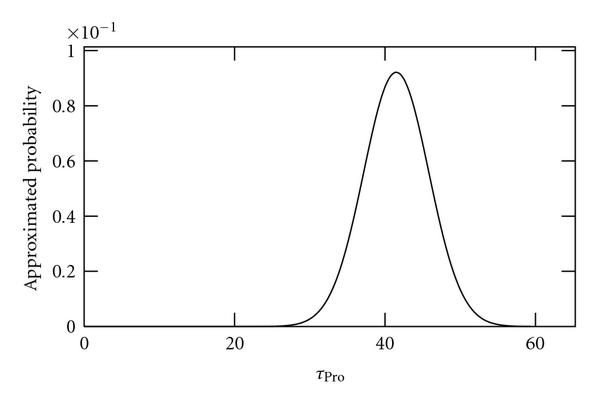
**Approximated probabilities for values of  inferred using simulated noiseless data from 100 cells**. The true value is 40 and the expected value is 41.5.

**Figure 4:**
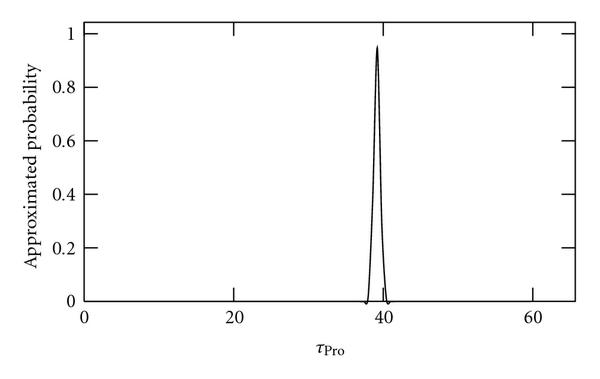
**Approximated probabilities for values of  inferred using simulated noiseless data from 1000 cells**. The true value is 40 and the expected value is 39.2.

We applied the method to sample sizes of 10, 100, and 1000 independent time series of length 2970 s (100 time points). As no external noise sources are applied to these data, we refer to it as "noiseless" data. Results are shown in Figures 2, 3, and 4, respectively. As seen, as the sample size is increased, the better becomes the inference of the true value of .

Interestingly, as seen from these results, using this method it is possible to show, even using a small sample size of 10, that the time length of the promoter open complex formation measurably affects the dynamics of RNA levels as previously shown by confronting numerical simulations with a null model [13].

**Figure 5:**
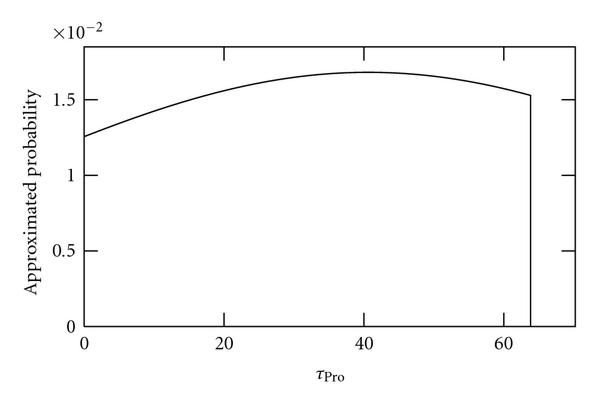
**Approximated probabilities for values of  inferred using simulated noisy data from 10 cells**. The true value is 40, the maximum likelihood value is 40.6 and the expected value is 32.9.

**Figure 6:**
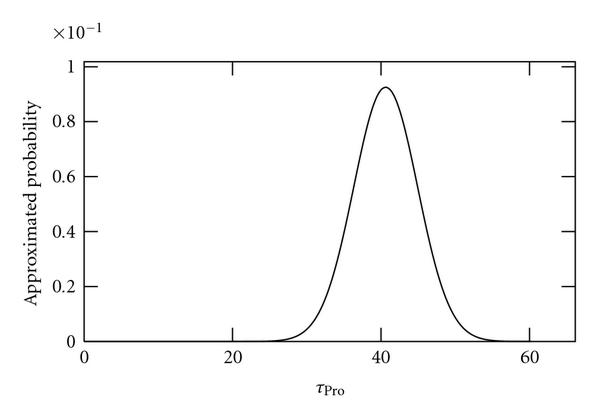
**Approximated probabilities for values of  inferred using simulated noisy data from 100 cells**. The true value is 40 and the expected value is 40.6.

**Figure 7:**
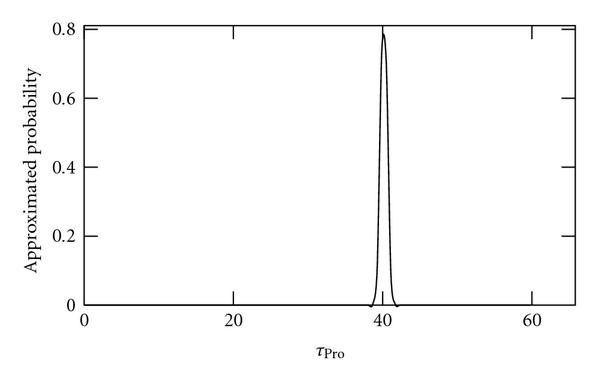
**Approximated probabilities for values of  inferred using simulated noisy data from 1000 cells**. The true value is 40 and the expected value is 40.6.

We now test the robustness of the method to experimental measurement error. For this, to the previous time series we add Gaussian noise "noisy data" as described in the Methods section. Results of the inference, using 10, 100 and 1000 time series, are shown in Figures 5, 6, and 7, respectively. As the results show, the accuracy of the method is not significantly affected when the standard deviation of the external noise is in the range 0 to 0.5. If the noise level in the data is increased beyond this, the results become biased.

Finally, we note that using 1000 time series for the inference procedure, the method takes 15 min to be completed on a contemporary personal computer.

## 4. Conclusions

We tested an inference method for inferring, from time series data, kinetic parameters affecting the dynamics of RNA levels subject to degradation. When inferring the duration of the promoter open complex formation, we showed that, for known values of the RNA degradation rate, the method is accurate and fast. When a reasonable amount of noise is added to the data the performance is not significantly affected.

The inference was shown possible when considering only one previous sample point, by approximating it with a time-homogeneous Markov chain. This is especially relevant as, in *E. coli*, most RNA mean levels are from 1 to a few [19], implying that the system may have very little memory of far past events.

While experimentally challenging, it is already possible to collect time series of RNA levels of living cells close to the accuracy assumed by the model. This can be done using a technique that is based on the ability of the MS2d-GFP protein complex to bind to a target RNA [20]. This system possesses some limitations, such as the need to maintain weak transcription rate so as to distinguish individual RNA molecules [10].

While the present approximative method proposed is still far from an analytical likelihood, it can serve as a crude statistical tool to analyze experimental time series data. In the future, we aim to extend this method to infer other kinetic parameters associated with the dynamics RNA and protein levels in prokaryotes. Also, we will apply this method to determine from real measurements of RNA levels, if these are influenced by currently unknown processes.
